# Control of Uterine Microenvironment by Foxp3^+^ Cells Facilitates Embryo Implantation

**DOI:** 10.3389/fimmu.2013.00158

**Published:** 2013-06-20

**Authors:** Ana Teles, Anne Schumacher, Marie-Cristine Kühnle, Nadja Linzke, Catharina Thuere, Peter Reichardt, Carlos Eduardo Tadokoro, Günter J. Hämmerling, Ana Claudia Zenclussen

**Affiliations:** ^1^Experimental Obstetrics and Gynecology, Medical Faculty, Otto-von-Guericke University Magdeburg, Magdeburg, Germany; ^2^PDBEB, Center for Neuroscience and Cell Biology, University of Coimbra, Coimbra, Portugal; ^3^Molecular Immunology, German Cancer Research (Center DKFZ), Heidelberg, Germany; ^4^Institute for Molecular and Clinical Immunology, Medical Faculty, Otto-von-Guericke University, Magdeburg, Germany; ^5^Instituto Gulbenkian de Ciências, Oeiras, Portugal

**Keywords:** regulatory T cells, implantation, pregnancy, fibrosis, inflammation

## Abstract

Implantation of the fertilized egg into the maternal uterus depends on the fine balance between inflammatory and anti-inflammatory processes. Whilst regulatory T cells (Tregs) are reportedly involved in protection of allogeneic fetuses against rejection by the maternal immune system, their role for pregnancy to establish, e.g., blastocyst implantation, is not clear. By using 2-photon imaging we show that Foxp3^+^ cells accumulated in the mouse uterus during the receptive phase of the estrus cycle. Seminal fluid further fostered Treg expansion. Depletion of Tregs in two Foxp3.DTR-based models prior to pairing drastically impaired implantation and resulted in infiltration of activated T effector cells as well as in uterine inflammation and fibrosis in both allogeneic and syngeneic mating combinations. Genetic deletion of the homing receptor CCR7 interfered with accumulation of Tregs in the uterus and implantation indicating that homing of Tregs to the uterus was mediated by CCR7. Our results demonstrate that Tregs play a critical role in embryo implantation by preventing the development of a hostile uterine microenvironment.

## Introduction

Normal pregnancy is a physiological state during which distinct processes take place at different stages. Pregnancy begins with the fertilization of the ovum, followed by implantation of the blastocyst in the maternal uterus. To implant, the blastocyst needs to adhere to the endometrium so that it can be provided with oxygen and nutrients. For these changes to occur tissue remodeling and inflammatory processes in the uterus are required. It has been observed that ablation of immunoregulatory molecules such as transforming growth factor beta (TGF-β) or heme oxygenase-1 (*Hmox1*) negatively interferes with implantation (McLennan and Koishi, 2004; Zenclussen et al., 2011). Thus, a balance between pro- and anti-inflammatory molecules is assumed to support successful implantation. Cells of the innate immune system present in the uterus are known to be critical for implantation. For example, uterine natural killer cells (uNK cells) produce IFN-γ, which contributes to the initiation of vascular modification and decidual integrity (Ashkar et al., 2000). Macrophages present in the uterus secrete IL-1, which is reportedly involved in implantation (Houser et al., 2011). Ablation of uterine dendritic cells (DC) causes implantation failure due to perturbed angiogenesis (Plaks et al., 2008) while the absence of uterine mast cells (MCs) leads to defective implantation and spiral artery remodeling (Woidacki et al., 2013).

Once implantation is established, a period of maintenance follows, during which the maternal immune system actively tolerates the foreign paternal antigens expressed by the fetus (Tafuri et al., 1995) while being fully active against potential infections. Maternal CD4^+^CD25^+^ regulatory T cells (Tregs) have been reported to contribute to the maintenance of tolerance during pregnancy by suppressing maternal alloreactive immune responses against paternal structures in fetal cells (Zenclussen et al., 2005; Shima et al., 2010; Rowe et al., 2012; Samstein et al., 2012). It has been speculated the Tregs also participate at initial stages of pregnancy, e.g., implantation (Zenclussen et al., 2005; Schumacher et al., 2007), at which inflammatory processes and innate cell populations play an important role. However, conclusive experimental proof that Tregs are important for implantation was missing. The present study addresses this question and by using specific models shows that Foxp3^+^ Tregs are needed for successful implantation because they prevent the development of a hostile uterine microenvironment. In their absence, inflammation and fibrosis occur, both negatively interfering with embryo implantation.

## Materials and Methods

### Study approval

All studies were conducted according to governmental and institutional regulations (AZ 42502-2-868, Magdeburg; AZ 35-9185.81/G-98/08, Heidelberg; AO 10/2010, Oeiras).

### Mice and Treg depletion

*Foxp3^*DTR*^* (Kim et al., 2007) and *Foxp3^*GFP*^* knock-in mice (Fontenot et al., 2005) were kindly provided by Alexander Rudensky (Washington, DC, USA) and Jocelyne Demengeot (Oeiras, Portugal) and bred in our facilities. CCR7^−*/*−^ mice were purchased from Jackson Laboratories; C57BL/6 and BALB/c (intact, vasectomized, and seminal vesicle-deficient) mice from Charles River, Germany. BAC transgenic Foxp3.LuciDTR-4 mice (Suffner et al., 2010) were generated and maintained at the DKFZ, Heidelberg. Mated females were checked daily for vaginal plugs; its appearance indicates day 0 of pregnancy. Females were mated and checked daily for vaginal plugs, its appearance indicates day 0.5 of pregnancy. Vaginal lavage was performed with 20 μL of 0.9% sodium chloride. Cycle stage was defined after observation of the cellular components under light microscopy (Axiovert C, Carl Zeiss, Germany; magnification ×200), and was confirmed by hematoxylin/Eosin. Treg depletion in Foxp3^*DTR*^ mice was performed by i.p. application of 15 ng/g body weight diphtheria toxin (DT) every fourth day. In Foxp3Luci.DTR mice, DT was applied daily beginning at day −2. Control mice received PBS or DT. Implantations sites at day 5 of pregnancy were identified after i.v. injection of 0.5% Chicago Blue dye.

### 2-Photon *in vivo* microscopy

Imagining of uterine *Foxp3^*GFP*^* cells during the estrus cycle was performed as follows. Animals were anesthetized by i.p. injection of ketamine and xylazine, 120 or 16 μg/g of mouse weight, respectively, and kept on a heating pad at 37°C. One of the uterine horns was carefully exposed and 0.1 M caffeine (Sigma-Aldrich, USA) was applied to decrease uterine contractions. For maternal blood visualization, animals were intravenously injected with 100 μl of Rhodamine B isothiocyanate-Dextran (RhoB-Dex) 70,000 KDa (Sigma-Aldrich, Inc., USA) before acquiring images in a multiphoton laser scanning microscope (MPLSM). We used a Prairie Ultima 2-photon microscope (Prairie Technologies, Inc.). The microscope was equipped with a Chameleon Ti:Sapphire laser (Coherent, Inc., USA), four top PMTs for simultaneous up to four channel acquisitions and a 20× water immersion objective (Olympus, Inc., USA). The laser was tuned to 880 nm to allow for concomitant excitation of RhoB-Dex and GFP^+^-Treg. The wavelength emission for RhoB-Dex and GFP is 590 and 509 nm, respectively. Sequential images were acquired for observation of Treg in the uterus. For analysis of uterine Treg images, we have developed our own software algorithms based on endogenous tissue markers information (e.g., location of blood vessels) from consecutive z-stacks acquisitions for stabilization of Treg movies. Once the images were stabilized, we used Imaris software (Bitplane AG, Inc.) for reconstruction of three-dimensional models in order to determine distribution in the uterus. For more detail, please see Zenclussen et al. (2013).

### Flow cytometry

The following fluorescence-conjugated antibodies were purchased from BD Biosciences or eBioscience: CD4 (RM-4 or RM4-5), CD8 (53-6.7), Foxp3 (FJK-16s or NRRF-30), Ki67 (B56), CD44 (IM7), CD62L (Mel-14). Samples were processed as described (Zenclussen et al., 2005; Woidacki et al., 2013), measured in a FACSCalibur or FACSCanto II (BD Biosciences) and analyzed with CellQuest Pro software (BD Biosciences).

### Histology and immunofluorescence

Analysis of uterine tissue was performed after staining with Hematoxylin/Eosin. The slides were dewaxed and left in Hematoxylin (Fluka Biochemika, Germany) for 1–2 min followed by rinse with tap water and a further incubation in Eosin (Fluka Biochemika, Germany) for 1–2 min and finally dehydrated to xylol and covered. Fibrosis extent was evaluated by Masson’s trichrome and Picrosirius red stainings. For the first, the dewaxed slides were left in picrosirius red stain solution (Sigma) for 1 h and rinsed in 10 dips of water before being dehydrated to xylol and covered. For Masson’s trichrome staining it was proceed as follows: after dewaxing, the slides were placed in Bouin’s solution (Sigma) overnight (ON) for mordating and washed in running tap water before staining for 5 min in working Weigert’s iron Hematoxylin solution (Sigma). After washing in running tap water, the samples were stained in Biebrich Scarlet-acid Fuchsin (Sigma) for 5 min and rinsed in distilled water. The slides were placed in Phosphomolybdic/Phosphotungstic acid solution (Sigma) for 10 min and the solution was discarded and substituted by aniline blue solution for five more min. Finally, the slides were placed in 1% acetic acid solution for 3 min and dehydrated to xylol before being covered.

Connective tissue growth factor (CtGF) in uterine tissue was studied by immunofluorescence. For this, tissue sections were dewaxed and blocked for 30 min with 3% bovine serum albumin (BSA) in PBS at room temperature (RT). The slides were then washed in PBS and incubated with anti-CTGF primary antibody (Abcam ab6992) in 2% BSA at a dilution of 1:200 ON at 4°C. After further washing the slides with PBS they were incubated with Alexa Fluor 488 goat anti-Rabbit IgG secondary antibody (Abcam) at a dilution of 1:200 for 45 min at RT. Sections were washed and the slides were covered with Dapi Vecta Shield mounting medium.

### *In vitro* proliferation

MACS-sorted Treg or effector T cells were stained with carboxyfluorescein diacetate succinimidyl ester (CFSE, Molecular Probes, Leiden, Netherlands), cultured with rmIL-2 (R & D systems) and treated with freshly prepared seminal vesicle fluid (SVF) from BALB/c males. Anti-TGF-β1 mAb was kindly provided by Hideo Yagita (Juntendo University, Tokyo, Japan).

### Quantitative real time PCR

mRNA was isolated using Trizol^®^ and cDNA synthesis was done using reverse transcriptase (Invitrogen, Karlsruhe, Germany). Real time PCR was performed with the help of an iCycler (BIORAD, Munich, Germany) using SYBR Green (Applied Biosystems, Germany). Initial denaturation was done at 95°C for 5 min followed by a denaturation step of 40 cycles of 45 s at 95°C. Annealing step followed at 59°C for 30 s 72°C for 30 s extension. The amount of cDNA was standardized with the reference gene β-actin. Primers are available upon request.

### Statistical analysis

Statistical analyses were performed with Prism software (GraphPad). Normality in the distribution of the data was analyzed by the D’Agostino and Pearson omnibus normality test. Differences in the percentage of CD4^+^Foxp3^*G*F*P*+^, CD8^+^ KI67^+^, CD44^+^, and CD62L^+^ cells between groups were analyzed by Mann–Whitney-*U* test. The *in vitro* experiments using cells cultured with SVF were analyzed by two-way ANOVA test. Implantation numbers were measured at day 5, data are expressed as medians of % of implanted females and analyzed by Fisher’s exact test. Levels of molecules measured by qPCR [IL-15, CCR5, CCL19, CXCL3, IL-1b, gp130, TNF-α, RORγτ, CCL5, CtGF, CXCL9, urokinase-type plasminogen activator (uPA), prostaglandin F receptor (Ptgfr), IL-9, Gal-1, leukemia inhibitory factor (LIF), p. 53] were analyzed by Mann–Whitney-*U* test. Number of animals used for each experiment is included in the Figure legends.

## Results

### Uterine Foxp3^+^ Tregs accumulate during the fertile phase of the cycle at the antimesometrial region

**Figure 1:**
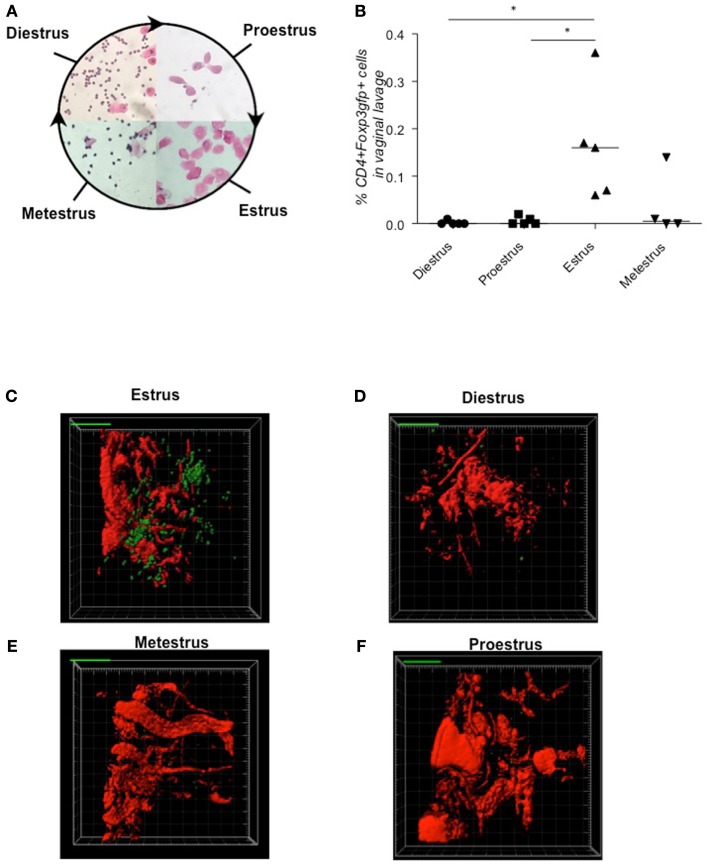
**Tregs fluctuate during estrus cycle peaking at estrus, time point of sexual receptivity**. Determination of the estrus cycle in non-pregnant females according to cell types in vaginal smears **(A)**. Percentage of CD4^+^Foxp3^*G*F*P*+^ cells of female Foxp3^*G*F*P*^ mice (*n* = 5/cycle stage) was analyzed in vaginal lavage **(B)**. Data are expressed as single dot plots with medians and were analyzed by Mann–Whitney-*U* test (*P ≤ 0.05). Snap shots of two-photon microscopy videos of Foxp3^*G*F*P*^ positive cells in uterus performed at different phases of the cycle **(C)** estrus phase; **(D)** diestrus phase; **(E)** metestrus phase; **(F)** proestrus phase.

First, we determined the frequencies of CD4^+^Foxp3^+^ Tregs in samples from *Foxp3^*gfp*^* female animals at each stage of the estrus cycle (diestrus, proestrus, estrus, metestrus, Figure 1A) by flow cytometry (Figure 1B). We observed substantial fluctuation in Treg frequency in the vaginal lavage, with Tregs peaking at the receptive phase, namely estrus, and decreasing toward metestrus. *In vivo* 2-photon microscopy in *Foxp3^*gfp*^* animals impressively demonstrated a clustering of Tregs in uterine tissue during estrus (Figure 1C; Movie S1 in Supplementary Material) that was not observed during the other phases of the estrus cycle, e.g., diestrus [Figure 1D; metestrus (Figure 1E) or proestrus (Figure 1F); Movies S2–S4 in Supplementary Material]. In agreement with these findings, previous work has suggested oscillations in uterine Foxp3 mRNA expression levels during the estrus cycle (Kallikourdis and Betz, 2007; Guerin et al., 2011). According to the 2-photon studies, Tregs seem to accumulate preferentially in the perivascular space close to small blood vessels at the antimesometrial region during estrus (Movie S1 in Supplementary Material).

### Seminal fluid fosters the expansion of uterine Foxp3^+^ Tregs

**Figure 2:**
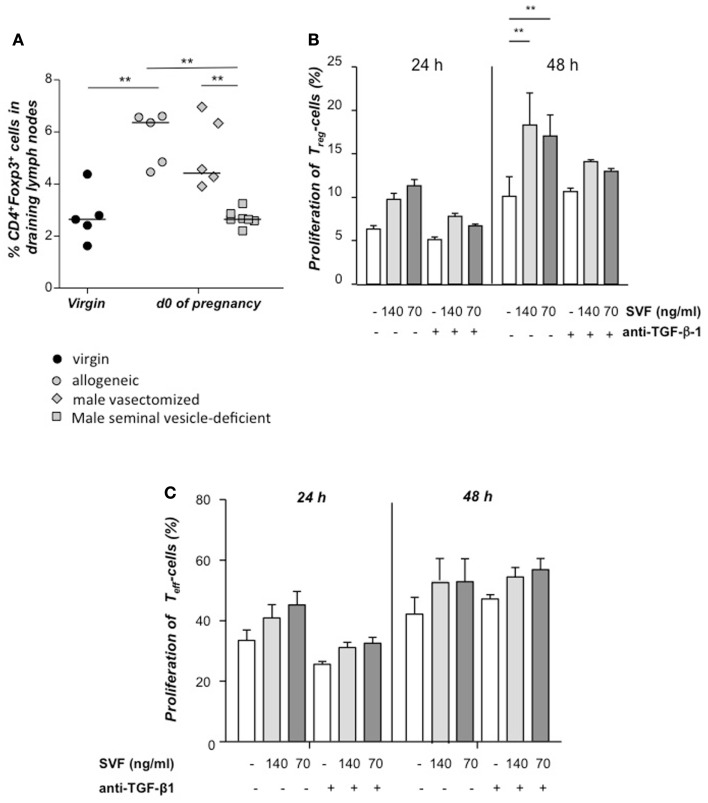
**Tregs expand *in vivo* and *in vitro* in presence of seminal fluid**. Percentage of CD4^+^Foxp3^+^ cells in the uterine draining lymph nodes of CBA/J females mated with seminal vesicle-deficient, vasectomized, or intact BALB/c males was analyzed at day of conception (day 0.5) and compared to virgin CBA/J females (*n* = 4–7) **(A)**. Statistical analysis were performed by Mann–Whitney-*U* test (**P ≤ 0.01). **(B)** Tregs were isolated from non-pregnant CBA/J females by magnetic cell sorting, stained with CFSE and cultured with seminal vesicle fluid (SVF) from BALB/c males. TGF-β1 was blocked with anti- TGF-β1 antibody, and proliferation of Treg determined after 24 h by using FACScan Calibur. Data are representative of four experiments and expressed as mean with SEM. Analysis was performed by two-way ANOVA test (**P ≤ 0.01). **(C)** Conventional T effector cells were isolated from non-pregnant CBA/J females by magnetic cell sorting, stained with CFSE and cultured with seminal vesicle fluid (SVF) from BALB/c males. TGF-β1 was blocked with anti- TGF-β1 antibody, and proliferation of Treg determined after 24 h by using FACScan Calibur. Data are representative of four experiments and expressed as mean with SEM. Analysis was performed by two-way ANOVA test and no statistically significant differences were found among the groups.

Next, we paired females with seminal vesicle-deficient, vasectomized, or intact allogeneic males. When compared to virgin mice, an expansion of Tregs was observed in the uterine draining lymph nodes from females paired with intact or vasectomized males, whereas no expansion was found in females mated with seminal vesicle-deficient males (Figure 2A). Hence, seminal fluid has a proliferative effect on Tregs *in vivo*. These results could be reproduced *in vitro* with CSFE-labeled CD4^+^CD25^+^ Tregs isolated by magnetic cell sorting, which were stimulated to proliferate by addition of seminal fluid and this was partially inhibited by addition of anti-TGF β (Figure 2B), whereas conventional T cells were not affected (Figure 2C). Thus, the accumulation of Tregs at sexual receptivity is followed by an expansion triggered by seminal fluid, most likely by TGF-β contained herein.

### Depletion of Foxp3^+^ Tregs prior to mating resulted in uterine inflammation and fibrosis that impaired implantation

**Figure 3:**
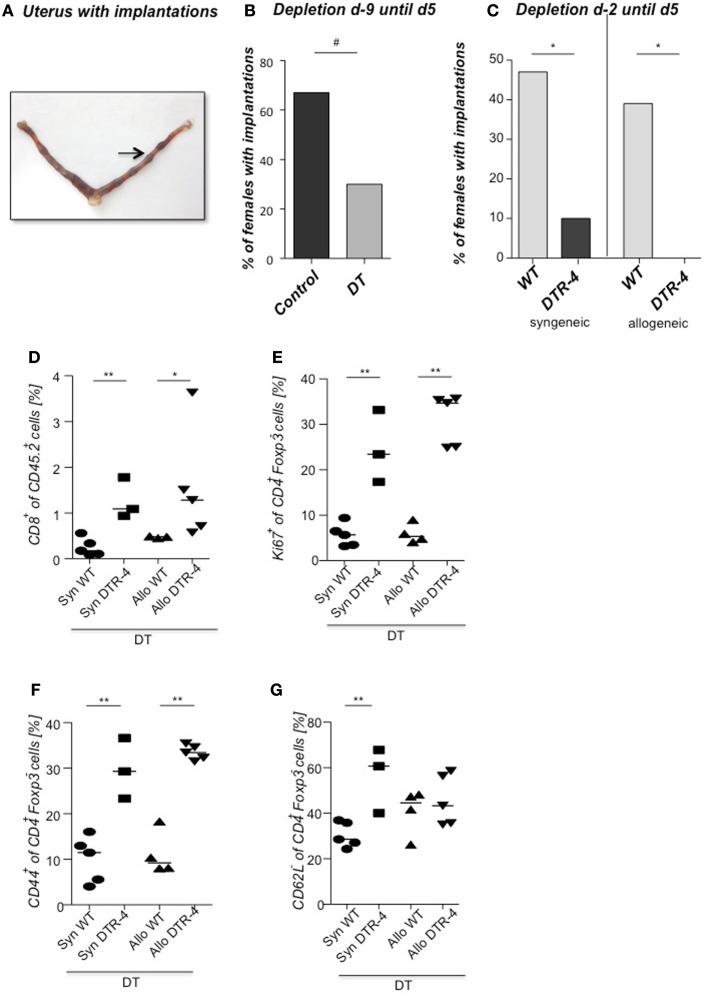
**Embryo implantation is impaired after Treg depletion**. Foxp3^+^ Treg were depleted in *Foxp3^*DTR*^* mice by application of DT every fourth day, starting 9 days before mating (day 9) with BALB/c males. Control groups received PBS. **(A)** shows a representative picture of a uterus stained at day 5 post conception with Chicago Blue dye application. The arrow indicates a representative implantation site. The percentage of females presenting implantations was analyzed on day 5 after mating [**(B)**, *n* = 5–12]. Foxp3^+^ Treg were depleted in Foxp3.LuciDTR-4 mice by daily application of DT, starting on day 2 with allogeneic (CBAJ) or syngeneic (C57/BL6) males. Control groups were wt C57/BL6 females treated with DT. Implantation numbers were measured at day 5 [**(C)**, *n* = 15–21]. For **(B,C)**, data are expressed as medians of % of implanted females and analyzed by Fisher’s exact test (#*P* < 0.1, *P < 0.05). Not all plugged animals became pregnant. In samples from animals shown in Figure 3B, the percentage of CD8^+^ cells was analyzed in the uterus by flow cytometry **(D)**, and the percentage of KI67^+^, CD44^+^, and CD62L^+^ **(E–G)** determined in uterine draining lymph nodes (*n* = 5/group). Data are expressed as medians and were analyzed by Mann–Whitney-*U* test (*P < 0.05; **P < 0.01).

**Figure 4:**
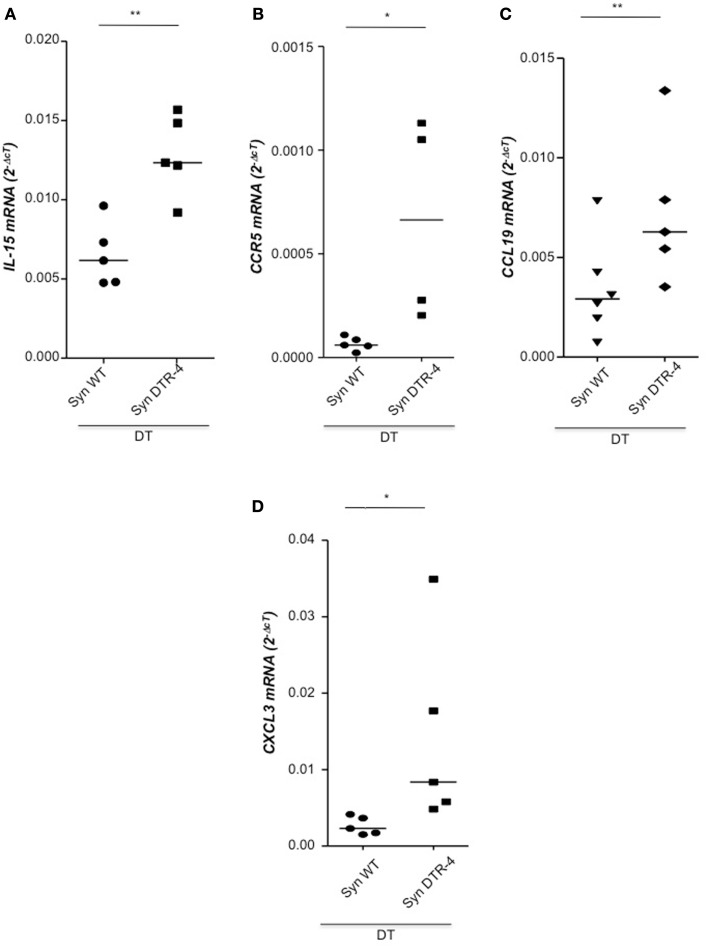
**Depletion of Tregs creates an inflammatory uterine milieu**. In samples from Foxp3.LuciDTR-4 or wild type animals treated with DT by daily application starting on day 2 and further mated syngeneically, inflammation markers were measured (*n* = 4–6/group) in uterine tissue by qPCR. Levels of IL-15 **(A)**, CCR5 **(B)**, CCL19 **(C)**, and CXCL3 **(D)** were significantly elevated in mice depleted of Tregs as compared to DT-treated controls. Data are expressed as single dots with medians and were analyzed by Mann–Whitney-*U* test (#*P* < 0.1, *P* < 0.05; **P < 0.01).

**Figure 5:**
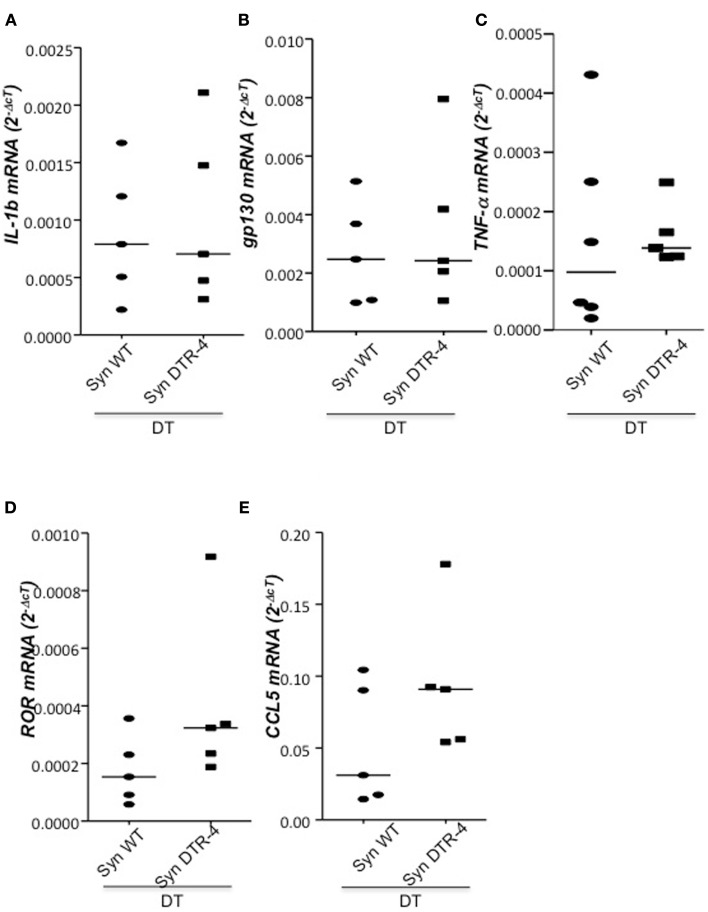
**Depletion of Tregs does not provoke changes in the levels of IL-1b, gp130, TNF-α, ROR-γτ, or CCL5**. In samples from Foxp3.LuciDTR-4 or wild type animals treated with DT by daily application starting on day 2 and further mated allogeneically, inflammation markers were measured (*n* = 4–6/group) in uterine tissue by qPCR. Levels of IL-1b **(A)**, gp130 **(B)**, TNF-α **(C)**, RORγτ **(D)**, and CCL5 **(E)** mRNA were comparable in mice depleted of Tregs and DT-treated controls. Data are expressed as single dot plots with medians and were analyzed by Mann–Whitney-*U* test. No statistically significant differences were observed between both groups in any of the molecules.

**Figure 6:**
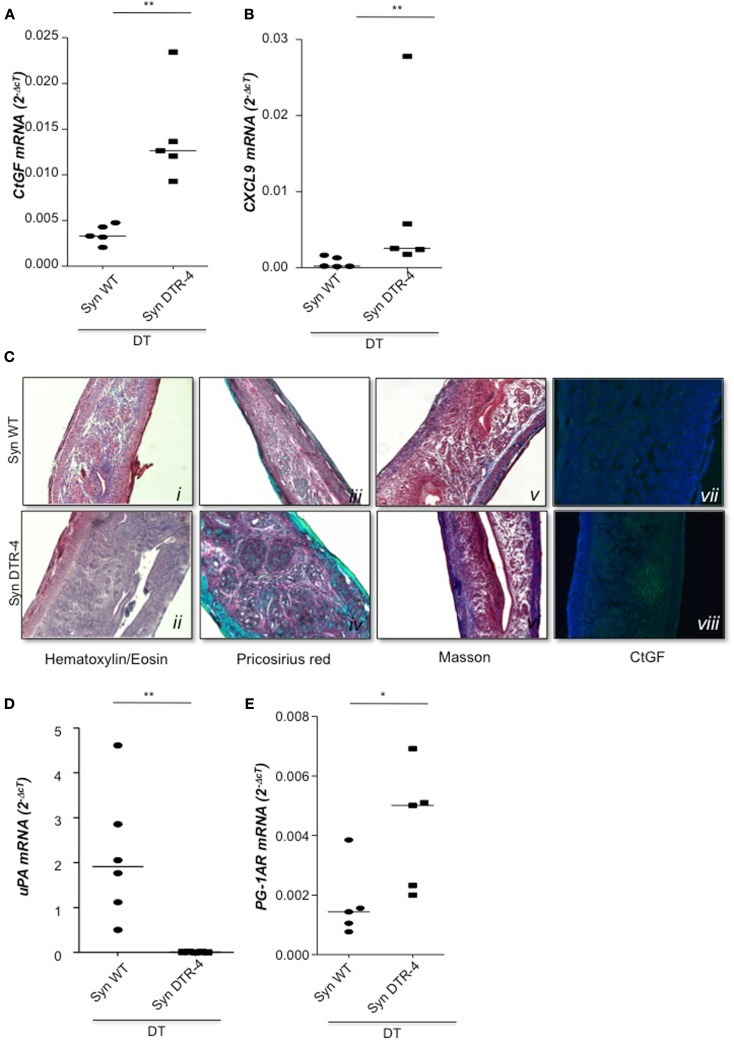
**Treg depletion leads to uterine fibrosis**. In samples from Foxp3.LuciDTR-4 or wild type animals treated with DT by daily application starting on day 2 and further mated syngeneically, fibrosis markers were measured (*n* = 4–6/group) in uterine tissue by qPCR. Levels of CtGF **(A)** and CXCL9 **(B)** were significantly elevated in mice depleted of Tregs as compared to DT-treated controls. Data are expressed as single dots with medians and were analyzed by Mann–Whitney-*U* test (#*P* < 0.1, **P < 0.01). Additionally, staining with Hematoxylin/Eosin revealed fibrosis areas with disorganized collagen fibers in animals without Tregs compared to wild type controls (**C** **ii** vs. **i**). Pricosirius red staining revealed a higher amount of green/blue-stained thin collagen fibers that confirms fibrosis in mice devoid of Tregs vs. controls (**C** **iv** vs. **iii**). Further, Masson’s staining manifested larger areas of collagen fibers in blue/violet in DT-treated Foxp3.Luci.DTR animals compared to DT-treated controls (**C** **vi** vs. **v**). Finally in (**C** **vii**, **viii**), immunofluorescence staining for CtGF confirmed accumulation of CtGF positive cells in mice depleted in Tregs as compared to the controls. All pictures were taken with a 10× objective. Analysis of uPA mRNA revealed that animals depleted in Tregs presented very low, almost undetectable levels of this molecule as compared to DT-treated controls **(D)**. Treg depletion further provoked an augmentation of prostaglandin 1A mRNA **(E)**.

**Figure 7:**
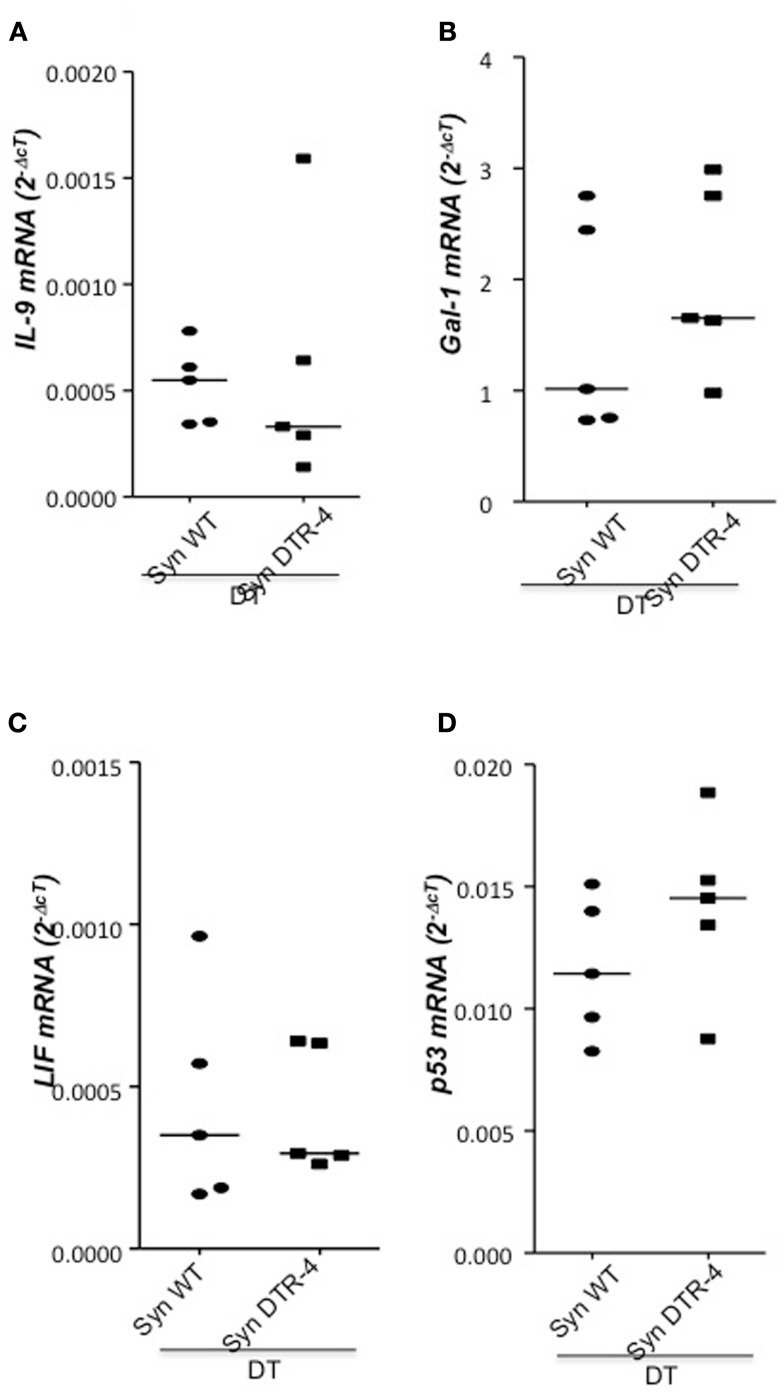
**Treg depletion does not provoke changes in IL-9, Gal-1, LIF, or p53**. In samples from Foxp3.LuciDTR-4 or wild type animals treated with DT by daily application starting on day 2 and further mated allogeneically, inflammation markers were measured (*n* = 4–6/group) in uterine tissue by qPCR. Levels of IL-9 **(A)**, Gal-1 **(B)**, LIF **(C)**, and p53 **(D)** mRNA were comparable in mice depleted of Tregs and DT-treated controls. Data are expressed as single dot plots with medians and were analyzed by Mann–Whitney-*U* test. No statistically significant differences were observed between both groups in any of the molecules.

As Treg accumulation at receptivity suggested a role for embryo implantation, we investigated the effect of CD4^+^Foxp3^+^ Treg depletion on implantation by using two distinct strains of Foxp3.DTR mice, namely Foxp3^*DTR*^ knock-in mice (Fontenot et al., 2005), and BAC-transgenic Foxp3.LuciDTR-4 mice (Suffner et al., 2010). Upon application of DT, at least 90% of CD4^+^Foxp3^+^ Tregs were specifically depleted in both strains. Thus, for Treg depletion Foxp3.DTR mice are superior to the frequently used anti-CD25 antibodies, which deplete only about 70% of CD4^+^Foxp3^+^ Treg, owing to limited expression of CD25 on Foxp3^+^ T cells (Shima et al., 2010). Moreover, Foxp3 represents a highly selective marker for Tregs, whereas CD25 is also expressed on a fraction of other leukocytes (Shima et al., 2010). Treg depletion was performed during various time intervals before mating, namely from day 9 to 5 of pregnancy, and from day 2 to 5. To confirm the occurrence of implantations at day 5, Chicago Blue was utilized (Figure 3A) Treg depletion beginning at day 9 in *Foxp3^*DTR*^* mice provoked a failure of the embryos to implant, whereas PBS- and DT-treated control mice displayed normal implantation (Figures 3B,C). When Foxp3.LuciDTR mice were used, syngeneic matings were included in order to elucidate whether the mechanisms leading to implantation failure were dependent on potentially present paternal alloantigens (Figure 3C). Treg ablation from day 2 to 5 resulted in severely impaired implantation not only in biologically relevant allogeneic pregnancies but also in syngeneic matings (Figure 3C). We speculated that at this early time point Tregs may counteract pro-inflammatory events occurring during implantation. Without Tregs, inflammation may be too strong and hinder the nidation of the embryo. Indeed, we observed that Treg depletion resulted in accumulation of CD8^+^ T cells in the uterus (Figure 3D) and the presence of activated T cells in lymph nodes draining the uterus, as indicated by the increased expression of KI67, CD44, and down-regulation of CD62L in CD4^+^Foxp3^−^ effector cells (Figures 3E–G). Additionally, the uterine tissue was inflamed and thickened after DT treatment. mRNA expression of the inflammatory mediators IL-15, CCR5, CCL19, and CXCL3, all inflammatory was upregulated (Figures 4A–D). Levels of IL-1β, gp130, TNF-α, ROR-gamma-t, or CCL5 were not significantly modified (Figures 5A–E). We also observed the appearance of fibrosis in the uterus of mice depleted of Treg as indicated by elevated levels of CtGF and CXCL9 mRNA (Figures 6A,B), important regulators of fibrosis (Gressner and Gressner, 2008; Zeremski et al., 2011). Fibrosis was further confirmed histologically in mice devoid of Tregs by means of Hematoxylin-Eosin (Figure 6Cii), Picrosirius (Figure 6Civ), and the Masson’s trichrome staining (Figure 6Cv) as well as by means of CtGF immunofluorescence (Figure 6Cviii) as compared to DT-treated control mice (Figures 6Ci,iii,v,vii). The levels of uPA, known to positively influence tissue remodeling and whose absence leads to infertility (Carmeliet et al., 1994), were downregulated and almost undetectable in the absence of Tregs (Figure 6D). It is of interest that Ptgfr was also upregulated (Figure 6E) as prostaglandin is routinely given to provoke therapeutic abortions (Wagner et al., 2011). No changes were found in the levels of the immunosuppressive factors IL-9 and Gal-1 (Figures 7A,B). LIF and p53, known to define fertility (Hu et al., 2007), were also not modified after Treg depletion (Figures 7C,D). Inflammation and fibrosis were only investigated in syngeneic matings in order to investigate the effects of Treg depletion independent of allogenicity. Together, the data indicate that depletion of Tregs prior to mating leads to a hostile uterine environment that is characterized by the occurrence of inflammation and fibrosis, thereby interfering with implantation.

### CCR7 mediated the homing of Treg to the uterus and its genetic ablation impaired implantation

The chemokine receptor CCR7 has been identified as a homing receptor on Tregs required for migration to lymph nodes and spleen (Worbs and Förster, 2007). In order to explore a role for CCR7 in Treg homing into the uterus and for embryo implantation, we employed CCR7-deficient mice. The number of CD4^+^Foxp3^+^ Treg in the uterus was drastically reduced in CCR7^−*/*−^ mice (Table 1), whereas the frequency of conventional CD4^+^Foxp3^−^ T cells was only partially reduced (data not shown). Consistent with the absence of uterine Treg, CCR7 deficiency resulted in implantation failure (Table 1). Supporting our findings on the importance of CCR7 on Treg for implantation, Guerin et al. (2011) reported the presence of CCL19, the ligand for CCR7, in glandular and luminal uterine epithelial cells, however a role for this chemokine for the recruitment of Treg into the uterus was not investigated. We show here that CCR7 mediates the homing of Treg to the uterus and that its absence hampers implantation.

**Table 1:** **Consequences of CCR7 deficiency in number of uterine Tregs and implantation rate**.

	% of uterine Foxp3^+^ cells within CD4 cells	Implantation rate
Wild type controls (*n* = 5)	11.97 (4.48–21.1)	100%
CCR7^−*/*−^animals (*n* = 5)	0 (0–0.01)	55%
Statistical significance	<0.05	<0.05

## Discussion

The presence of Foxp3^+^ Tregs in uterus during early pregnancy has already been reported (Schumacher et al., 2007; Thuere et al., 2007). Here, by employing flow cytometry, we confirm that these cells are present in the uterus in the non-pregnant state and that they further fluctuate during the different phases of the estrous cycle. It seems that this fluctuation occurs in response to hormones, as has been proposed after observing variations in the Foxp3 mRNA levels (Kallikourdis and Betz, 2007; Guerin et al., 2011). *In vivo* 2-photon microscopy in *Foxp3^*gfp*^* animals impressively confirmed oscillations in the frequency of Treg in the uterus and further revealed a clustering of Tregs in uterine tissue during the receptive phase of the cycle, the estrus. This may be interpreted as an accumulation of these cells at the future implantation niches and preparation of the uterus for implantation.

We further observed that Foxp3^+^ Tregs expanded immediately after copulation at day 0 of pregnancy. The expansion occurred after mating females with intact or vasectomized males but not after mating with males lacking seminal vesicles, suggesting that components of the seminal fluid are active in expanding Tregs which is in accordance with other reports (Robertson et al., 2009; Guerin et al., 2011). Treg expansion by seminal fluid could be confirmed by *in vitro* experiments that further indicated TGF-β as a possible modulator of Treg expansion. Hence, Treg presence at the moment of pairing and their further expansion by paternal components implies a role for embryo implantation.

To investigate the potential role of CD4^+^Foxp3^+^ Tregs we employed two distinct strains of Foxp3.DTR mice for Treg depletion, namely Foxp3^*DTR*^ knock-in mice and BAC-transgenic Foxp3.LuciDTR-4 mice. We observed that depletion of Tregs prior to mating resulted in a dramatic impairment of implantation. In line with this, it was recently reported that diminished endometrial Foxp3 mRNA was associated with infertility in women (Jasper et al., 2006). We speculated that at this early time point Tregs may counteract pro-inflammatory events occurring during implantation and facilitate a uterine environment that supports implantation. Without Tregs, inflammation may be too strong and may favor a hostile uterine microenvironment that would hinder nidation of the embryo. Indeed, we observed a strong upregulation of the pro-inflammatory mediators IL-15, CCR5 CCL19 and CXCL3. We also found augmented levels of CD8^+^ cells and activated CD4^+^ cells in uterus. These findings are supported by studies showing that Treg depletion in Foxp3.DTR mice leads to T cell activation and pro-inflammatory modulation of the microenvironment of sites where Tregs accumulate, such as tumors (Li et al., 2010). Besides inflammation and accumulation of effector cells, we observed swollen uteruses that were devoid of implantations. The development of uterine fibrosis was indicated by the upregulation of CtGF and CXCL9. uPA, a positive regulator of tissue remodeling in the uterus was almost undetectable in the absence of Treg, which is in line with impaired infertility of uPA deficient animals (Carmeliet et al., 1994). Our data reveals that the lack of Tregs results in uterine conditions that are hostile for the embryo to implant. The levels of the known fertility factors LIF and p53 (Hu et al., 2007) were not modified in the absence of Treg.

We conclude that Treg are pivotal for implantation as they control the uterine microenvironment needed for the blastocyst to attach and grow in the uterus. Many of the effects seen here after Treg depletion are reminiscent of clinical parameters associated with infertility. Pathologies like endometritis, endometriosis, inflammatory pelvic diseases, and fallopian tube blockage associated with inflammatory processes cause the metamorphosis of reproductive tract tissues and vaginal fluid and are often a cause of infertility by hindering nidation (Shah et al., 2005; He et al., 2010; Braundmeier et al., 2012). Likewise, it is known that, e.g., pelvic inflammation, uterine swelling, and intraluminal occluding fibrosis of the oviduct after infections with Chlamydia sp. are associated with infertility (Weiss et al., 2009; Wiesenfeld et al., 2012). Thus, it seems that the specific depletion of Treg mimics alterations induced by pathogens that lead to infertility, specifically an inflammatory milieu and fibrosis, thereby hindering successful implantation.

The targets for regulation by uterine Tregs are not clear, but as T cell-deficient mice such as RAG mutant mice lack both Tregs and effector T cells, but present normal implantation rates, it seems reasonable to assume that effector T cells are major targets and that uterine Tregs serve to control excessive production of inflammatory cytokines. We also demonstrated that homing of Tregs to the uterus depends on CCR7 and that CCR7 deficiency resulted in implantation failure. In support of this finding, Guerin et al. (2011) described the presence of CCL19, the ligand for CCR7, in glandular and luminal uterine epithelial cells.

In another study it was reported that Treg depletion with anti-CD25 antibody at day 2.5 post conception would hinder implantation only in allogeneic but not syngeneic mating combinations (Shima et al., 2010). At first sight this appears to be at disagreement with our findings that Treg depletion in Foxp3.DTR mice causes implantation failure also in syngeneic matings. However, in view of the fact that Foxp3.DTR allow more efficient and more specific depletion of Tregs the above authors themselves have suggested the use of Foxp3.DTR mice for respective studies, as has been done here (Shima et al., 2010).

Once pregnancy is established, the embryo expressing paternal antigens needs to be protected against the maternal immune system. In several reports Tregs have been shown to be important for this process (Zenclussen et al., 2005; Schumacher et al., 2007; Samstein et al., 2012). Using the same Foxp3.DTR knock-in mice employed here, Samstein et al. (2012) have shown that the *de novo* conversion of Treg in the periphery contributes to embryo protection. However, as only 10% of all embryos are rejected in the absence of Foxp3^+^ Tregs their protective role for maintaining the integrity of the fetus appears to be limited as compared to the strong effect of Treg on implantation observed here. Additional and multiple mechanisms must exist to ensure maternal tolerance toward the fetus. Several distinct tolerance mechanisms have been reported, such as awareness of maternal T cells for paternal alloantigens and acquisition of a transient state of tolerance during pregnancy (Tafuri et al., 1995), ignorance of fetus-specific T cells, epigenetic silencing in decidual tissue of chemokines that attract T cells (Nancy et al., 2012), and others.

In view of the importance of pregnancy for survival of a species; its first phase, namely embryo implantation, must be tightly controlled. Our data reveal the importance of Treg for this process, likely by suppressing excessive inflammation in the uterine microenvironment. The results are relevant for human pregnancies, especially when designing protocols for improving fertility.

## Conflict of Interest Statement

The authors declare that the research was conducted in the absence of any commercial or financial relationships that could be construed as a potential conflict of interest.

## Supplementary Material

The Supplementary Material for this article can be found online at: http://www.frontiersin.org/Mucosal_Immunity/10.3389/fimmu.2013.00158/abstract

Supplementary Movie S1 **Estrus: 3D reconstruction of live 2-photon-microscopy of uterus from a representative Foxp3^*gfp*^ animal during the estrus phase oft he estrus cycle**. The green cells are Tregs clustered at the mesometrial region. Animals were anesthetized by i.p. injection of ketamine and xylazine, 120 or 16 μg/g of mouse weight, respectively, and kept on a heating pad at 37°C. One of the uterine horns was carefully exposed and 0.1 M caffeine (Sigma-Aldrich, USA) was applied to decrease uterine contractions. For maternal blood visualization, animals were intravenously injected with 100 μl of Rhodamine B isothiocyanate-Dextran (RhoB-Dex) 70,000 KDa (Sigma-Aldrich, Inc., USA) before acquiring images in a multiphoton laser scanning microscope (MPLSM). We used a Prairie Ultima 2-photon microscope (Prairie Technologies, Inc.). The microscope was equipped with a Chameleon Ti:Sapphire laser (Coherent, Inc., USA), four top PMTs for simultaneous up to four channel acquisitions and a 20× water immersion objective (Olympus, Inc., USA). The laser was tuned to 880 nm to allow for concomitant excitation of RhoB-Dex and GFP^+^-Treg. The wavelength emission for RhoB-Dex and GFP is 590 and 509 nm, respectively. Sequential images were acquired for observation of Treg in the uterus. For analysis of uterine Treg images, we have developed our own software algorithms based on endogenous tissue markers information (e.g., location of blood vessels) from consecutive z-stacks acquisitions for stabilization of Treg movies. Once the images were stabilized, we used Imaris software (Bitplane AG, Inc.) for reconstruction of three-dimensional models in order to determine distribution in the uterus.

Supplementary Movie S2 **Diestrus: depicts a 3D reconstruction of representative live 2-photon-microscopy of uterus from a Foxp3^*gfp*^ animal at diestrus**. Green Tregs are scarce. Animals were anesthetized by i.p. injection of ketamine and xylazine, 120 or 16 μg/g of mouse weight, respectively, and kept on a heating pad at 37°C. One of the uterine horns was carefully exposed and 0.1 M caffeine (Sigma-Aldrich, USA) was applied to decrease uterine contractions. For maternal blood visualization, animals were intravenously injected with 100 μl of Rhodamine B isothiocyanate-Dextran (RhoB-Dex) 70,000 KDa (Sigma-Aldrich, Inc., USA) before acquiring images in a multiphoton laser scanning microscope (MPLSM). We used a Prairie Ultima 2-photon microscope (Prairie Technologies, Inc.). The microscope was equipped with a Chameleon Ti:Sapphire laser (Coherent, Inc., USA), four top PMTs for simultaneous up to four channel acquisitions and a 20× water immersion objective (Olympus, Inc., USA). The laser was tuned to 880 nm to allow for concomitant excitation of RhoB-Dex and GFP^+^-Treg. The wavelength emission for RhoB-Dex and GFP is 590 and 509 nm, respectively. Sequential images were acquired for observation of Treg in the uterus. For analysis of uterine Treg images, we have developed our own software algorithms based on endogenous tissue markers information (e.g., location of blood vessels) from consecutive z-stacks acquisitions for stabilization of Treg movies. Once the images were stabilized, we used Imaris software (Bitplane AG, Inc.) for reconstruction of three-dimensional models in order to determine distribution in the uterus.

Supplementary Movie S3 **Metestrus: shows a video from a 3D reconstruction of live 2-photon microscopy showing almost undetectable green Tregs within the uterus**. Animals were anesthetized by i.p. injection of ketamine and xylazine, 120 or 16 μg/g of mouse weight, respectively, and kept on a heating pad at 37°C. One of the uterine horns was carefully exposed and 0.1 M caffeine (Sigma-Aldrich, USA) was applied to decrease uterine contractions. For maternal blood visualization, animals were intravenously injected with 100 μl of Rhodamine B isothiocyanate-Dextran (RhoB-Dex) 70,000 KDa (Sigma-Aldrich, Inc., USA) before acquiring images in a multiphoton laser scanning microscope (MPLSM). We used a Prairie Ultima 2-photon microscope (Prairie Technologies, Inc.). The microscope was equipped with a Chameleon Ti:Sapphire laser (Coherent, Inc., USA), four top PMTs for simultaneous up to four channel acquisitions and a 20× water immersion objective (Olympus, Inc., USA). The laser was tuned to 880 nm to allow for concomitant excitation of RhoB-Dex and GFP^+^-Treg. The wavelength emission for RhoB-Dex and GFP is 590 and 509 nm, respectively. Sequential images were acquired for observation of Treg in the uterus. For analysis of uterine Treg images, we have developed our own software algorithms based on endogenous tissue markers information (e.g., location of blood vessels) from consecutive z-stacks acquisitions for stabilization of Treg movies. Once the images were stabilized, we used Imaris software (Bitplane AG, Inc.) for reconstruction of three-dimensional models in order to determine distribution in the uterus.

Supplementary Movie S4 **Proestrus: distribution of green Foxp3^*gfp*+^ cells (Tregs) inside the uterus during proestrus**. Animals were anesthetized by i.p. injection of ketamine and xylazine, 120 or 16 μg/g of mouse weight, respectively, and kept on a heating pad at 37°C. One of the uterine horns was carefully exposed and 0.1 M caffeine (Sigma-Aldrich, USA) was applied to decrease uterine contractions. For maternal blood visualization, animals were intravenously injected with 100 μl of Rhodamine B isothiocyanate-Dextran (RhoB-Dex) 70,000 KDa (Sigma-Aldrich, Inc., USA) before acquiring images in a multiphoton laser scanning microscope (MPLSM). We used a Prairie Ultima 2-photon microscope (Prairie Technologies, Inc.). The microscope was equipped with a Chameleon Ti:Sapphire laser (Coherent, Inc., USA), four top PMTs for simultaneous up to four channel acquisitions and a 20× water immersion objective (Olympus, Inc., USA). The laser was tuned to 880 nm to allow for concomitant excitation of RhoB-Dex and GFP^+^-Treg. The wavelength emission for RhoB-Dex and GFP is 590 and 509 nm, respectively. Sequential images were acquired for observation of Treg in the uterus. For analysis of uterine Treg images, we have developed our own software algorithms based on endogenous tissue markers information (e.g., location of blood vessels) from consecutive z-stacks acquisitions for stabilization of Treg movies. Once the images were stabilized, we used Imaris software (Bitplane AG, Inc.) for reconstruction of three-dimensional models in order to determine distribution in the uterus.

## References

[B1] Ashkar A. A. Di Santo J. P. Croy B. A. (2000). Interferon gamma contributes to initiation of uterine vascular modification, decidual integrity, and uterine natural killer cell maturation during normal murine pregnancy. J. Exp. Med. 192, 259–270 10.1084/jem.192.2.259 10899912 PMC2193246

[B2] Braundmeier A. Jackson K. Hastings J. Koehler J. Nowak R. Fazleabas A. (2012). Induction of endometriosis alters the peripheral and endometrial regulatory T cell population in the non-human primate. Hum. Reprod. 27, 1712–1722 10.1093/humrep/des083 22442246 PMC3357193

[B3] Carmeliet P. Schoonjas L. Kieckens L. Ream B. Degen J. Bronson R. (1994). Physiological consequences of loss of plasminogen activator gene function in mice. Nature 368, 419–424 10.1038/368419a0 8133887

[B4] Fontenot J. D. Rasmunssen J. P. Gavin M. A. Rudensky A. Y. (2005). A function for interleukin-2 in Foxp3-expressing regulatory T cells. Nat. Immunol. 6, 1142–1151 10.1038/ni1263 16227984

[B5] Gressner O. A. Gressner A. M. (2008). Connective tissue growth factor: a fibrogenic master switch in fibrotic liver diseases. Liver Int. 28, 1065–1079 10.1111/j.1478-3231.2008.01826.x 18783549

[B6] Guerin L. R. Moldenhauer L. M. Prins J. R. Bromfield J. J. Hayball J. D. Robertson S. A. (2011). Seminal fluid regulates accumulation of FOXP3+ regulatory T cells in the preimplantation mouse uterus through expanding the FOXP3+ cell pool and CCL19-mediated recruitment. Biol. Reprod. 85, 397–408 10.1095/biolreprod.110.088591 21389340

[B7] He Q. Tsang L. L. Ajonuma L. C. Chan H. C. (2010). Abnormally up-regulated cystic fibrosis transmembrane conductance regulator expression and uterine fluid accumulation contribute to *Chlamydia trachomatis*-induced female infertility. Fertil. Steril. 93, 2608–2614 10.1016/j.fertnstert.2010.01.040 20227074

[B8] Houser B. L. Tilburgs T. Hill J. Nicotra M. L. Strominger J. L. (2011). Two unique human decidual macrophage populations. J. Immunol. 186, 2633–2642 10.4049/jimmunol.1003153 21257965 PMC3712354

[B9] Hu W. Feng Z. Teresky A. K. Levine A. J. (2007). p53 regulates maternal reproduction through LIF. Nature 450, 721–724 10.1038/nature05993 18046411

[B10] Jasper M. J. Tremellen K. P. Robertson S. A. (2006). Primary unexplained infertility is associated with reduced expression of the T-regulatory cell transcription factor Foxp3 in endometrial tissue. Mol. Hum. Reprod. 12, 301–308 10.1093/molehr/gal032 16574699

[B11] Kallikourdis M. Betz A. G. (2007). Periodic accumulation of regulatory T cells in the uterus: preparation for the implantation of a semi-allogeneic fetus? PLoS ONE 2:e382 10.1371/journal.pone.0000382 17440618 PMC1847704

[B12] Kim J. M. Rasmussen J. P. Rudensky A. Y. (2007). Regulatory T cells prevent catastrophic autoimmunity throughout the lifespan of mice. Nat. Immunol. 8, 191–197 10.1038/ni1428 17136045

[B13] Li X. Kostareli E. Suffner J. Garbi N. Hämmerling G. J. (2010). Efficient Treg depletion induces T-cell infiltration and rejection of large tumors. Eur. J. Immunol. 40, 3325–3335 10.1002/eji.201041093 21072887

[B14] McLennan I. S. Koishi K. (2004). Fetal and maternal transforming growth factor-beta 1 may combine to maintain pregnancy in mice. Biol. Reprod. 70, 1614–1618 10.1095/biolreprod.103.026179 14766723

[B15] Nancy P. Tagliani E. Tay C. S. Asp P. Levy D. E. Erlebacher A. (2012). Chemokine gene silencing in decidual stromal cells limits T cell access to the maternal-fetal interface. Science 336, 1317–1321 10.1126/science.1220030 22679098 PMC3727649

[B16] Plaks V. Birnberg T. Berkutzki T. Sela S. BenYashar A. Kalchenko V. (2008). Uterine DCs are crucial for decidua formation during embryo implantation in mice. J. Clin. Invest. 118, 3954–3965 10.1172/JCI36682 19033665 PMC2582932

[B17] Robertson S. A. Guerin L. R. Bromfield J. J. Branson K. M. Ahlström A. C. Care A. S. (2009). Seminal fluid drives expansion of the CD4+CD25+ T regulatory cell pool and induces tolerance to paternal alloantigens in mice. Biol. Reprod. 80, 1036–1045 10.1095/biolreprod.108.074658 19164169 PMC2849830

[B18] Rowe J. H. Ertelt J. M. Xin L. Way S. S. (2012). Pregnancy imprints regulatory memory that sustains anergy to fetal antigen. Nature 490, 102–106 10.1038/nature11462 23023128 PMC3465465

[B19] Samstein R. M. Josefowicz S. Z. Arvey A. Treuting P. M. Rudensky A. Y. (2012). Extrathymic generation of regulatory T cells in placental mammals mitigates maternal-fetal conflict. Cell 150, 29–38 10.1016/j.cell.2012.05.031 22770213 PMC3422629

[B20] Schumacher A. Wafula P. O. Bertoja A. Z. Sollwedel A. Thuere C. Wollenberg I. (2007). Mechanisms of action of regulatory T cells specific for paternal antigens during pregnancy. Obstet. Gynecol. 110, 1137–1145 10.1097/01.AOG.0000284625.10175.31 17978130

[B21] Shah A. A. Schripsema J. H. Imtiaz M. T. Sigar I. M. Kasimos J. Matos P. G. (2005). Histopathologic changes related to fibrotic oviduct occlusion after genital tract infection of mice with *Chlamydia muridarum*. Sex. Transm. Dis. 32, 49–56 10.1097/01.olq.0000148299.14513.11 15614121

[B22] Shima T. Sasaki Y. Itoh M. Nakashima A. Ishii N. Sugamura K. (2010). Regulatory T cells are necessary for implantation and maintenance of early pregnancy but not late pregnancy in allogeneic mice. J. Reprod. Immunol. 85, 121–129 10.1016/j.jri 20439117

[B23] Suffner J. Hochweller K. Kühnle M. C. Li X. Kroczek R. A. Garbi N. (2010). Dendritic cells support homeostatic expansion of Foxp3+ regulatory T cells in Foxp3.LuciDTR mice. J. Immunol. 184, 1810–1820 10.4049/jimmunol.0902420 20083650

[B24] Tafuri A. Alferink J. Möller P. Hämmerling G. J. Arnold B. (1995). T cell awareness of paternal alloantigens during pregnancy. Science 270, 630–633 10.1126/science.270.5236.630 7570020

[B25] Thuere C. Zenclussen M. L. Schumacher A. Langwisch S. Schulte-Wrede U. Teles A. (2007). Kinetics of regulatory T cells during murine pregnancy. Am. J. Reprod. Immunol. 58, 514–523 10.1111/j.1600-0897.2007.00538.x 17997750

[B26] Wagner N. Abele H. Hoopmann M. Grischke E. M. Blumenstock G. Wallwiener D. (2011). Factors influencing the duration of late first and second-trimester termination of pregnancy with prostaglandin derivates. Eur. J. Obstet. Gynecol. Reprod. Biol. 155, 75–78 10.1016/j.ejogrb.2010.10.019 21112135

[B27] Weiss G. Goldsmith L. T. Taylor R. N. Bellet D. Taylor H. S. (2009). Inflammation in reproductive disorders. Reprod. Sci. 16, 216–229 10.1177/1933719108330087 19208790 PMC3107847

[B28] Wiesenfeld H. C. Hillier S. L. Meyn L. A. Amortegui A. J. Sweet R. L. (2012). Subclinical pelvic inflammatory disease and infertility. Obstet. Gynecol. 120, 37–43 10.1097/AOG.0b013e31825a6bc9 22678036

[B29] Woidacki K. Popovic M. Metz M. Schumacher A. Linzke N. Teles A. (2013). Mast cells rescue implantation defects caused by c-kit deficiency. Cell Death Dis. 4, e462 10.1038/cddis.2012.214 23328669 PMC3564001

[B30] Worbs T. Förster R. (2007). A key role for CCR7 in establishing central and peripheral tolerance. Trends Immunol. 28, 274–280 10.1016/j.it.2007.04.002 17462953

[B31] Zenclussen A. C. Gerlof K. Zenclussen M. L. Sollwedel A. Bertoja A. Z. Ritter T. (2005). Abnormal T cell reactivity against paternal antigens in spontaneous abortion: adoptive transfer of pregnancy-induced CD4+CD25+ T regulatory cells prevents fetal rejection in a murine abortion model. Am. J. Pathol. 166, 811–822 10.1016/S0002-9440(10)62302-4 15743793 PMC1602357

[B32] Zenclussen A. C. Olivieri D. N. Dustin M. L. Tadokoro C. E. (2013). In vivo multiphoton microscopy technique to reveal the physiology of the mouse uterus. Am. J. Reprod. Immunol. 69, 281–289 10.1111/aji.12066 23279099

[B33] Zenclussen M. L. Casalis P. A. El-Mousleh T. Rebelo S. Langwisch S. Linzke N. (2011). Haem oxygenase-1 dictates intrauterine fetal survival in mice via carbon monoxide. J. Pathol. 225, 293–304 10.1002/path.2946 21744344

[B34] Zeremski M. Dimova R. Astemborski J. Thomas D. L. Talal A. H. (2011). CXCL9 and CXCL10 chemokines as predictors of liver fibrosis in a cohort of primarily African-American injection drug users with chronic hepatitis C. J. Infect. Dis. 204, 832–836 10.1093/infdis/jir424 21849280 PMC3156920

